# Cluster analysis integrating age and body temperature for mortality in patients with sepsis: a multicenter retrospective study

**DOI:** 10.1038/s41598-022-05088-z

**Published:** 2022-01-20

**Authors:** Moon Seong Baek, Jong Ho Kim, Young Suk Kwon

**Affiliations:** 1 Department of Internal Medicine, Chung-Ang University Hospital, Chung-Ang University College of Medicine, Seoul, Republic of Korea; 2 Department of Anesthesiology and Pain Medicine, College of Medicine, Chuncheon Sacred Heart Hospital, Hallym University, 77 Sakju-ro, Chuncheon, 24253 South Korea; 3 Institute of New Frontier Research Team, Hallym University, Chuncheon, South Korea

**Keywords:** Diseases, Signs and symptoms

## Abstract

It is not clear whether mortality is associated with body temperature (BT) in older sepsis patients. This study aimed to evaluate the mortality rates in sepsis patients according to age and BT and identify the risk factors for mortality. We investigated the clusters using a machine learning method based on a combination of age and BT, and identified the mortality rates according to these clusters. This retrospective multicenter study was conducted at five hospitals in Korea. Data of sepsis patients aged ≥ 18 years who were admitted to the intensive care unit between January 1, 2011 and April 30, 2021 were collected. BT was divided into three groups (hypothermia < 36 °C, normothermia 36‒38 °C, and hyperthermia > 38 °C), and age groups were divided using a 75-year age threshold. Kaplan‒Meier analysis was performed to assess the cumulative mortality over 90 days. A K-means clustering algorithm using age and BT was used to characterize phenotypes. During the study period, 15,574 sepsis patients were enrolled. Overall, 90-day mortality was 20.5%. Kaplan‒Meier survival analyses demonstrated that 90-day mortality rates were 27.4%, 19.6%, and 11.9% in the hypothermia, normothermia, and hyperthermia groups, respectively, in those ≥ 75 years old (Log-rank *p* < 0.001). Cluster analysis demonstrated three groups: Cluster A (relatively older age and lower BT), Cluster B (relatively younger age and wide range of BT), and Cluster C (relatively higher BT than Cluster A). Kaplan‒Meier curve analysis showed that the 90-day mortality rates of Cluster A was significantly higher than those of Clusters B and C (24.2%, 17.1%, and 17.0%, respectively; Log-rank *p* < 0.001). The 90-day mortality rate correlated inversely with BT groups among sepsis patients in either age group (< 75 and ≥ 75 years). Clustering analysis revealed that the mortality rate was higher in the cluster of patients with relatively older age and lower BT.

## Introduction

Sepsis is defined as life-threatening organ dysfunction caused by a dysregulated host response to infection^1^. A scoring system based on the signs of systemic inflammatory response syndrome (SIRS has been found to be inadequate for identification of sepsis^2^. Thus, the quick Sequential Organ Failure Assessment (qSOFA) score was introduced to recognize patients who are likely to have sepsis early. However, criteria such as fever are still widely used in the diagnosis of infection. Additionally, body temperature (BT) is an accepted prognostic factor in sepsis patients. Several studies have reported that mortality rates were lower in patients with hyperthermia and higher in those with hypothermia^3–7^.

Age is another factor affecting mortality in patients with sepsis. In a prospective observational study, patients ≥ 80 years had higher in-hospital mortality than patients aged 65–79 years (54.2% vs. 47.4%, *p* = 0.02)^8^. Furthermore, Shimazui et al. investigated the implications of BT in sepsis patients according to age^9^. They found that, in patients < 75 years, the risk of 90-day mortality was 1.7 times higher for those with BT < 36 °C than for those with BT ≥ 36 °C (*p* = 0.025). On the other hand, BT did not affect mortality in patients ≥ 75 years. Park et al. further subdivided sepsis into three BT groups, i.e., hypothermic (< 36 °C), normothermic (36–38 °C), and hyperthermic (> 38 °C) groups. In-hospital mortality rates and BT were inversely correlated (30.8%, 20.6%, and 8.5% in the three BT groups, respectively; *p* < 0.001)^5^. However, mortality rates according to these three BT groups have not been investigated in older sepsis patient.

There are distinct patient subclasses or endotypes in sepsis because the host response to infection is heterogeneous^10^. Clusters of multi-organ dysfunction syndrome or subphenotypes have been reported using BT trajectories in patients with sepsis^11,12^. Recently, Zhanga et al. suggested two classes of sepsis with different immunosuppression and mortality rates, using deep learning-based clustering^13^. We hypothesized that clusters based on a combination of age and BT may exist, and that machine learning would be helpful for identifying such clusters. This study aimed to evaluate the mortality rates in sepsis patients according to age and BT and identify the risk factors for mortality. We investigated the clusters using a machine learning method based on a combination of age and BT, and identified the mortality rates according to these clusters.

## Results

### Patient characteristics

**Figure 1:**
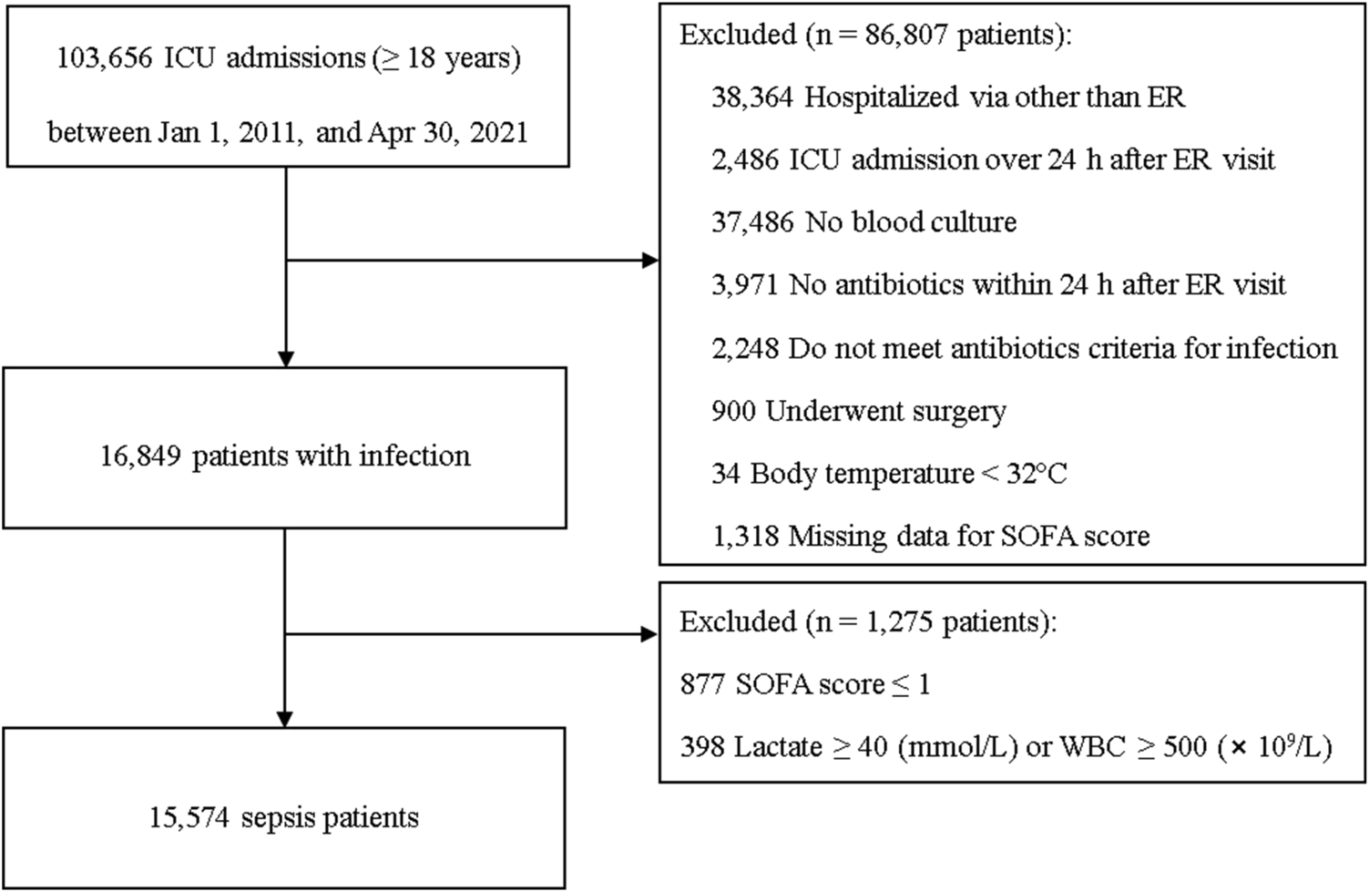
Flow chart of the patients. ICU = intensive care unit; ER = emergency room; SOFA = Sequential Organ Failure assessment; and WBC = white blood cell.

During the study period, 103,656 patients aged ≥ 18 years were admitted to intensive care unit (ICU) (Fig. 1). We excluded 86,807 patients who did not meet the inclusion criteria as follows: hospitalization via departments other than the emergency room (ER; n = 38,364), ICU admission more than 24 h after the ER visit (n = 2486), no blood culture (n = 37,486), no antibiotics treatment within 24 h after ER visit (n = 3971), antibiotics criteria for infection not met (n = 2248), surgery during hospitalization (n = 900), BT < 32 °C (n = 34), and missing data for the SOFA score (n = 1318). Then, 16,849 patients with infection were enrolled. Among them, 877 patients who had SOFA score ≤ 1 and 398 patients with outlier lactate and WBC values were excluded. The remaining 15,574 sepsis patients were enrolled.

Baseline characteristics of sepsis patients according to mortality are presented in Table 1. The mean age of the patients was 70.3 years (± 14.9), and their mean SOFA score was 6.5 (± 3.3). Septic shock occurred in 29.2% of the patients and mechanical ventilation was applied in 33.6% of the patients. Overall, 90-day mortality was 20.5% (n = 3190). The mean BT at admission was 37.0 °C (± 1.1). Overall, 9.7% of patients were classified as hypothermic, 73.6% as normothermic, and 16.8% as hyperthermic.

**Table 1:** Baseline characteristics of sepsis patients according to mortality.

Variables	Total (n = 15,574)	90-day survivor (n = 12,384)	90-day non-survivor (n = 3190)	*p* value
Age (years)	70.3 ± 14.9	69.8 ± 15.1	72.4 ± 13.9	< 0.001
Male sex (%)	8976 (57.6)	7040 (56.8)	1936 (60.7)	< 0.001
Body mass index (kg/m^2^)	22.0 ± 4.2	22.2 ± 4.2	21.2 ± 4.1	< 0.001
SIRS	1.9 ± 1.0	1.8 ± 1.0	2.0 ± 1.0	< 0.001
qSOFA	1.5 ± 0.8	1.4 ± 0.8	1.7 ± 0.8	< 0.001
SOFA score	6.5 ± 3.3	6.1 ± 3.1	8.1 ± 3.6	< 0.001
APACHE II score	19.8 ± 7.0	18.9 ± 6.6	23.1 ± 7.4	< 0.001
Charlson comorbidity index	5.3 ± 2.7	5.3 ± 2.7	5.5 ± 2.6	< 0.001
**Comorbidities (%)**				
Hypertension	9100 (58.4)	7319 (59.1)	1781 (55.8)	0.001
Diabetes	6123 (39.3)	4958 (40.0)	1165 (36.5)	< 0.001
Cardiac disease	4842 (31.1)	3899 (31.5)	943 (29.6)	0.036
Chronic lung disease	3062 (19.7)	2503 (20.2)	559 (17.5)	0.001
Chronic renal disease	2452 (15.7)	1959 (15.8)	493 (15.5)	0.614
Chronic liver disease	2106 (13.5)	1702 (13.7)	404 (12.7)	0.112
Cerebrovascular disease	4664 (29.9)	3802 (30.7)	862 (27.0)	< 0.001
Solid tumor	3289 (21.1)	2407 (19.4)	882 (27.6)	< 0.001
Hematologic malignancy	252 (1.6)	173 (1.4)	79 (2.5)	< 0.001
**Vital signs**				
Systolic blood pressure (mmHg)	120 ± 34	122 ± 34	115 ± 34	< 0.001
Diastolic blood pressure (mmHg)	72 ± 21	72 ± 21	69 ± 21	< 0.001
Respiratory rate (per minute)	23 ± 6	23 ± 6	24 ± 6	0.001
Heart rate (per minute)	83 ± 20	83 ± 19	84 ± 22	< 0.001
Body temperature (°C)	37.0 ± 1.1	37.0 ± 1.1	36.8 ± 1.1	< 0.001
Glasgow coma scale	11.1 ± 4.3	11.5 ± 4.1	9.3 ± 4.7	< 0.001
**Subgroup of body temperature (%)**				< 0.001
< 36 (°C)	1504 (9.7)	1082 (8.7)	422 (13.2)	
36–38 (°C)	11,455 (73.6)	9081 (73.3)	2374 (74.4)	
> 38 (°C)	2615 (16.8)	2221 (17.9)	394 (12.4)	
Septic shock (%)	4545 (29.2)	3103 (25.1)	1442 (45.2)	< 0.001
**Laboratory findings**				
White blood cell count (10^9^/L)	13.1 ± 8.9	12.9 ± 7.5	13.7 ± 12.8	0.002
Hemoglobin (g/dL)	11.7 ± 2.7	11.8 ± 2.7	11.2 ± 2.8	< 0.001
Platelet (10^9^/L)	220 ± 122	223 ± 121	208 ± 126	< 0.001
Creatinine (mg/dL)	1.9 ± 2.2	1.9 ± 2.2	2.1 ± 2.1	< 0.001
C-reactive protein (mg/L)	99 ± 100	95 ± 99	112 ± 101	< 0.001
Lactic acid (mmol/L)	3.9 ± 3.8	3.5 ± 3.5	5.2 ± 4.6	< 0.001
Mechanical ventilation (%)	5235 (33.6)	3547 (28.6)	1688 (52.9)	< 0.001
CRRT (%)	2195 (14.1)	1363 (11.0)	832 (26.1)	< 0.001
Vasopressor use (%)	5702 (36.6)	4097 (33.1)	1605 (50.3)	< 0.001
Corticosteroid use (%)	2514 (16.1)	1802 (14.6)	712 (22.3)	< 0.001
Transfusion (%)	3129 (20.1)	2250 (18.2)	879 (27.6)	< 0.001
Combination antibiotic therapy (%)	9621 (61.8)	7492 (60.5)	2129 (66.7)	< 0.001
Length of stay (days)	18.5 ± 22.0	19.7 ± 23.4	13.7 ± 14.8	< 0.001
ICU stay (days)	9.3 ± 14.1	9.1 ± 14.7	9.9 ± 11.5	0.001
Duration of MV (days)	4.1 ± 11.0	3.6 ± 11.0	6.2 ± 9.6	< 0.001

Values are presented as mean ± SD, or n (%).

SIRS, systemic inflammatory response syndrome; SOFA, sequential organ failure assessment; APACHE, acute physiology and chronic health evaluation; CRRT, continuous renal replacement therapy; ICU, intensive care unit; and MV, mechanical ventilation.

Compared to survivors, non-survivors were older (69.8 ± 15.1 years vs. 72.4 ± 13.9 years, *p* < 0.001) and had a higher SOFA score. Septic shock (25.1% vs. 45.2%, *p* < 0.001), mechanical ventilation, and use of vasopressors were more common in non-survivors than in survivors. Hypothermia was more common in non-survivors than in survivors (13.2% vs. 8.7%), but hyperthermia was common in survivors than in non-survivors (17.9% vs. 12.4%, *p* < 0.001).

### Risk factors for 90-day mortality in sepsis patients

In the Cox proportional univariate analysis, age, male, higher SOFA and APACHE II scores, higher CCI, hypo- and normothermia, septic shock, mechanical ventilation, CRRT, and vasopressor, corticosteroid, transfusion, and combination antibiotics therapy were significantly associated with 90-day mortality (Table 2). In multivariable analysis, patients aged ≥ 75 years had 1.43 times higher 90-day mortality than those aged < 75 years (HR 1.428 [95% CI 1.328–1.536], *p* < 0.001). Compared to hyperthermia, normothermia and hypothermia were significantly associated with 90-day mortality (normothermia: HR 1.353, 95% CI 1.214–1.507 and hypothermia: HR 1.231, 95% CI 1.067–1.421, *p* = 0.001). Furthermore, male sex, higher SOFA and APACHE II scores, mechanical ventilation, CRRT, and vasopressor, corticosteroid, transfusion, and combination antibiotics therapy use were significant risk factors for 90-day mortality.

**Table 2:** Cox’s proportional analyses of variables associated with 90-day mortality.

Variables	Univariate analysis	*P* value	Multivariable analysis	*P* value
	HR (95% CI)		HR (95% CI)	
**Age (years)**		< 0.001		< 0.001
< 75	Reference		Reference	
≥ 75	1.273 (1.187–1.365)		1.428 (1.328–1.536)	
**Sex**		< 0.001		0.023
Female	Reference		Reference	
Male	1.146 (1.067–1.230)		1.088 (1.012 1.171)	
SOFA score	1.170 (1.159–1.181)	< 0.001	1.074 (1.055–1.093)	< 0.001
APACHE II score	1.077 (1.072–1.081)	< 0.001	1.024 (1.017–1.031)	< 0.001
Charlson comorbidity index	1.020 (1.007–1.003)	0.002		
**Subgroup of body temperature**		< 0.001		< 0.001
> 38 °C	Reference		Reference	
36–38 °C	1.402 (1.276–1.580)	< 0.001	1.353 (1.214–1.507)	< 0.001
< 36 °C	2.094 (1.825–2.402)	< 0.001	1.231 (1.067–1.421)	0.004
Septic shock	2.288 (2.134–2.454)	< 0.001	1.613 (1.366 1.904)	< 0.001
Mechanical ventilation	2.564 (2.392–2.749)	< 0.001	1.464 (1.340–1.599)	< 0.001
CRRT	2.554 (2.360–2.764)	< 0.001	1.425 (1.302–1.560)	< 0.001
Vasopressor use	1.935 (1.805–2.074)	< 0.001	0.676 (0.570 0.802)	< 0.001
Corticosteroid use	1.584 (1.457–1.721)	< 0.001	1.215 (1.115–1.324)	< 0.001
Transfusion	1.621 (1.500–1.752)	< 0.001	1.251(1.153–1.358)	< 0.001
Combination antibiotic therapy	1.266 (1.176–1.362)	< 0.001	1.180 (1.095–1.272)	< 0.001

HR, hazard ratio; CI, confidence interval; SOFA, sequential organ failure assessment; APACHE, acute physiology and chronic health evaluation; and CRRT, continuous renal replacement therapy.

### Kaplan‒Meier curve analysis for 90-day mortality

**Figure 2:**
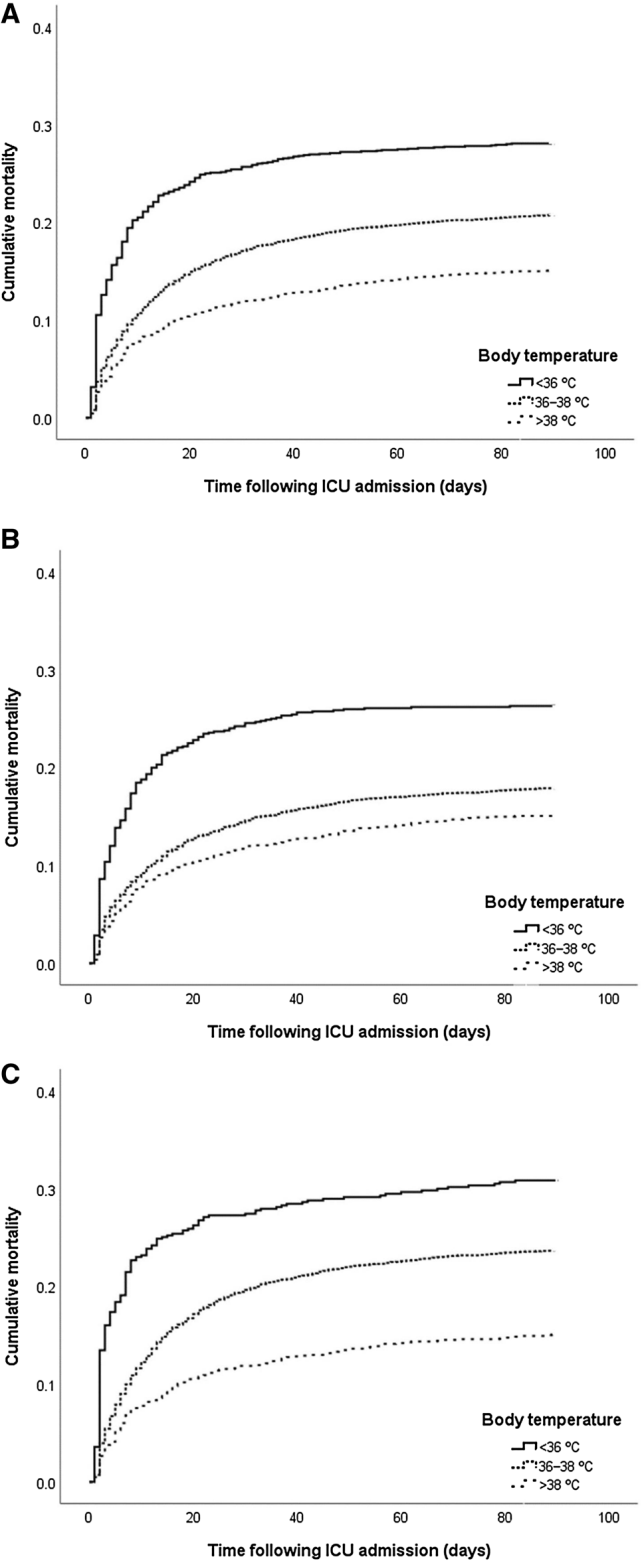
Probability of cumulative mortality according to the body temperature category in sepsis. (**A**) Overall patients (Log-rank *p* < 0.001). (**B**) Patients aged < 75 years (Log-rank *p* < 0.001). (**C**) Patients aged ≥ 75 years (Log-rank *p* < 0.001).

Kaplan‒Meier survival analyses for 90-day mortality rates are shown in Fig. 2. In the sepsis patients overall, 90-day mortality rates were 28.1% in the hypothermia group, 20.7% in the normothermia group, and 15.1% in the hyperthermia group (Log-rank *p* < 0.001). In those younger than 75 years, 90-day mortality rates were 24.5% in the hypothermia group, 14.6% in the normothermia group, and 12.0% in the hyperthermia group (Log-rank *p* < 0.001). In those older than 75 years, 90-day mortality rates were 27.4% in the hypothermia group, 19.6% in the normothermia group, and 11.9% in the hyperthermia group (Log-rank *p* < 0.001).

### Clustering analysis

**Figure 3:**
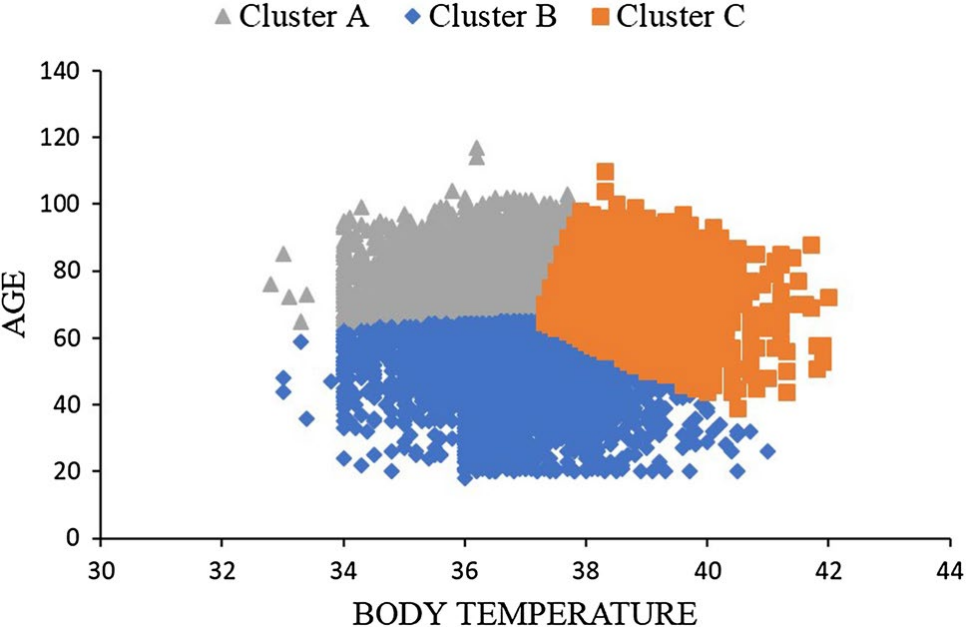
Distribution of the three patient clusters defined by age and body temperature.

**Figure 4:**
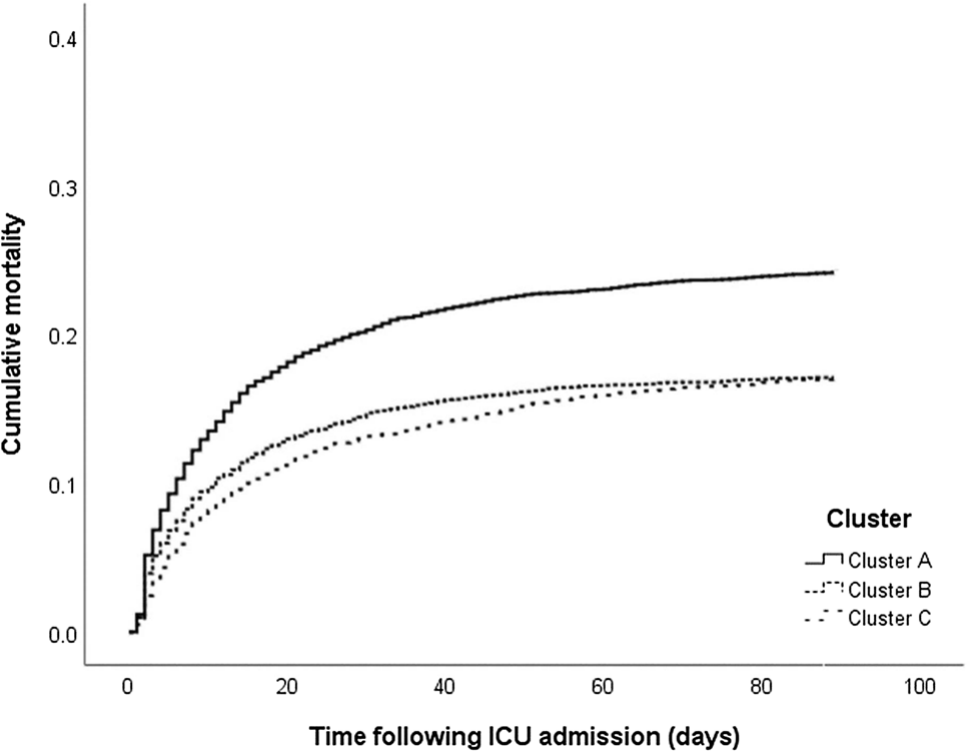
Probability of cumulative mortality according to the three clusters in patients with sepsis (Log-rank *p* < 0.001).

According to clustering method, sepsis patients were divided into three groups using age and BT as variables (Additional File 2 and Fig. 3). Age and BT differed significantly among the three clusters: Cluster A, Cluster B, and Cluster C. The mean age was 79.1 years (± 7.3) in Cluster A, 50.9 years (± 10.4) in Cluster B, and 74.5 years (± 9.4) in Cluster C (*p* < 0.001). BT was 36.5 °C (± 0.7) in Cluster A, 36.6 °C (± 1.0) in Cluster B, and 38.2 °C (± 0.8) in Cluster C (*p* < 0.001). Kaplan‒Meier curve analysis showed that the 90-day mortality rate in Cluster A was significantly lower than those in Cluster B and C (24.2% in Cluster A, 17.1% in Cluster B, and 17.0% in Cluster C, Log-rank *p* < 0.001) (Fig. 4).

## Discussion

This multicenter retrospective study revealed an association between age and BT in sepsis patients. The hypothermia group showed the highest 90-day mortality rate, and the mortality was lowest in the hyperthermia group. The trend of the mortality rate was similar in both the < 75-years and ≥ 75-years age-groups. Cox proportional analyses showed that older age and BT were significantly associated with mortality. Moreover, the clustering analysis demonstrated that the mortality rate was higher in the older age group with lower BT than in those with older age and higher BT as well as in the younger age group.

In agreement with a previous study, our analysis showed that the 90-day mortality rate was inversely correlated with BT groups among sepsis patients^5–7^. Hyperthermia was perceived as an adaptive physiological response, whereas hypothermia was thought to be associated with poor outcomes because it was a maladaptive response^14^. However, these thermoregulatory manifestations are recognized as the results of adaptation in cases of different sepsis severities^15^. Romanovsky et al. suggested that fever is a disease-fighting strategy in the mild to moderate phase, and facilitates pathogen clearance^16^. Hypothermia represents the late phase, where the disease has already progressed; therefore, its aim is energy-saving. In this regard, our results provided evidence of an association of BT and mortality, and revealed the prognostic implication of BT in sepsis.

In our study, a negative correlation between BT and mortality was observed in both the < 75-years and ≥ 75-years age-groups, consistently. Inconsistent with our results, Shimazui et al. have reported that BT alterations are not associated with mortality in older sepsis patients, whereas such an association was found in those who were younger^9^. They argued that this was the result of a blunted host inflammatory response. Vital signs, including BT, change with advancement in age^17^, in which older individuals have a lower baseline BT^18^, and thermoregulatory responses can be impaired^19^. Altered thermoregulatory responses in older individuals can be explained by reduced heat production capacity caused by reduced muscle mass, impaired peripheral vasoconstriction response, or reduced fat mass, resulting in increased heat loss^18–22^. Fever can result in early recognition of sepsis, leading to immediate commencement of antibiotics therapy. Moreover, sepsis patients who present with normothermia and hypothermia have a lower compliance with sepsis care bundles than patients with hyperthermia^5^. In experimental studies, hyperthermia is associated with inhibition of parasite growth and antimicrobial susceptibility among bacteria^23,24^. Therefore, we suggest that lower BT in older sepsis patients is associated with a worse prognosis. Our results are supported by those of previous studies, which showed that hypothermia is a significant predictor of mortality in sepsis patients older than 65 years^25,26^.

Normal BT is considered to be 36.8 °C, although it ranges from 35.6 to 38.2 °C^27^, and fever is diagnosed at a BT ≥ 38.3 °C^28^. Nonetheless, these BT threshold values differ across studies^4,5,7,9,29,30^. In addition, classification of the older population using the age cutoff of 75 years may be considered arbitrary. Moreover, because core BT is decreased with age, fever in older patients can be a more significant finding than it is in younger patients^31^. In this regard, our results, the association of mortality and clusters integrated with age and BT by machine learning methods, suggest several implications for sepsis. Although sepsis patients were divided into three clusters, Cluster A, which included patients with relatively older age and lower BT, showed significantly higher mortality rates than the other clusters. Furthermore, there was no significant difference in mortality between Cluster B, including patients with relatively younger age and wide range of BT, and Cluster C, comprising individuals with relatively higher BT than those in Cluster A. These findings indicated that age and BT play a complex role in the mortality of sepsis and patients with relatively older age and hypothermia have a higher mortality.

Recently, unsupervised cluster analysis has been reported to identify the phenotypes of study populations with heterogeneous characteristics: ICU patients^32^, sepsis patients^11^, and critically ill COVID-19 patients^33^. To the best of our knowledge, no previous study has applied cluster analysis to characterize phenotypes based on BT and age that are associated with mortality in sepsis patients. These results present further insights into the relationship between age and BT in sepsis.

Our multicenter study had several limitations. First, this was a retrospective study, and the sepsis cohort was created by an operational definition of sepsis using electronic medical records. Therefore, misclassification of sepsis was possible. However, the sepsis criteria proposed by Rhee et al.^34^ are widely used in many cohort studies, and the mortality rates reported for the Korean population were similar to our results^5,35^. Second, the information for out-of-hospital mortality within 90 days in discharged patients was not presented. Third, data for BT were collected from the vital signs routinely recorded for triage in the ER. Bhavani suggested that there are various sepsis phenotypes according to the BT trajectory within the first 72 h, and that this differs between survivors and non-survivors^12^. Therefore, the initial BT may not accurately reflect the early phase of BT, because it may change over time. Owing to these potential limitations for generalizability, further prospective studies are needed.

In conclusion, the 90-day mortality rate was inversely correlated with BT groups among sepsis patients. This negative correlation between BT and mortality was observed in both the < 75-year and ≥ 75-year age-groups. Clustering analysis revealed that the mortality rate was higher in the cluster of patients with relatively older age and lower BT. These results suggest that age and BT have a complex effect on the outcome of sepsis. Thus, sepsis patients with older age and hypothermia should be examined more carefully at presentation.

## Materials and methods

### Study design and patients

This multicenter study was conducted at 5 university-affiliated hospitals in the Republic of Korea. Hallym University Medical Center comprises hospitals in different provinces (two in Seoul, two in Gyeonggi, and one in Gangwon), and adopted the Clinical Data Warehouse system for extraction of electronic medical records. Data of patients aged ≥ 18 years who were admitted to the ICU between January 1, 2011, and April 30, 2021, were collected retrospectively. To set first records of ER as the index time, we enrolled patients who were admitted to the ICU via the ER. Hence, patients were excluded if they were admitted via departments other than the ER, were admitted more than 24 h after an ER visit, did not fulfill the diagnostic criteria for sepsis, or had missing values or outlier values.

The retrospective study protocol was approved by the Institutional Review Board of Chuncheon Sacred Hospital (CHUNCHEON 2021–09-004), which waived the requirement for informed consent. All procedures in this study were performed according to the relevant guidelines and regulations.

### Data collection

The following information was extracted within 24 h of presentation: age, sex, body mass index, SIRS, qSOFA, Sequential Organ Failure Assessment (SOFA) score, Acute Physiology and Chronic Health Evaluation (APACHE) II score, Charlson Comorbidity Index (CCI), comorbidities, main diagnosis, vital signs, laboratory results with arterial blood gases, mechanical ventilation use, continuous renal replacement therapy (CRRT), and vasopressor, corticosteroid, transfusion, or antibiotic use. Variables related to outcomes included 90-day mortality, length of hospital stay, ICU stay, and duration of mechanical ventilation.

### Diagnosis and definitions

All ICU patients admitted via the ER were screened. The time of diagnosis was determined based on the first records of vital signs at ER presentation. Patients with infection were considered if they met the following criteria: the presence of an order for blood culture, intravenous antibiotics administration within 24 h of presentation, and administration of antibiotics for at least 4 consecutive days (hospital stay ≥ 4 days), or continuation of antibiotics until 1 day before death or discharge (hospital stay ≤ 3 days)^34^. Sepsis and septic shock were defined by the Sepsis-3 criteria^1^. Sepsis was defined by a SOFA score ≥ 2 in patients who fulfilled infection criteria. Septic shock was defined by use of vasopressors and lactate level > 2 mmol/L on the day of presentation.

The qSOFA score was defined by the sum of three variables: respiratory rate ≥ 2, systolic blood pressure ≤ 100, and Glasgow coma scale < 15. SIRS was defined by the sum of four variables: BT > 38 °C or < 36 °C, heart rate > 90 beats/min, respiratory rate > 20/min or PCO2 < 32 mmHg, white blood cells > 12,000/μl or < 4000/μl, or band > 10%^36^. BT subgroups were created by classifying hypothermia as < 36 °C, normothermia as 36‒38 °C, and hyperthermia as > 38°C^5^. Age was divided into two subgroups based on a cut-off of 75 years (< 75 years and ≥ 75 years)^9^. We excluded non-physiological temperature data (temperatures < 32 °C and > 44 °C), with reference to a prior publication^37^.

### Clustering analysis

K-means clustering algorithm was used for clustering. This clustering algorithm is used for grouping data into a number of k clusters. This is a type of unsupervised machine learning, and can be used to identify homogeneous subgroups from unlabeled input data^38^. The k-means clustering algorithm groups the given data into k clusters and minimizes the variance of the difference between each cluster and distance^39^. This is a type of self-learning algorithm and is responsible for labeling unlabeled input data. In this study, the analysis was conducted as follows. First, k (number of clusters) data objects from a set of data objects D, containing 10 data objects, was randomly extracted. Then, these data objects were set as the centroid of each cluster (default setting). For each data object in set D, the distance from the k cluster centroid objects was computed, and the centroid of each data object was found with the highest similarity. Each data object was then assigned to the center point obtained. Then, the center point of the cluster was recalculated based on the clusters reassigned in step 2. Steps 2 and 3 were repeated until the cluster belonging to each data object did not change. Assuming that the center of the *i*-th cluster is *μ* and the set of points belonging to the cluster is S, the overall variance is calculated as follows:

$$ V = \mathop \sum \limits_{i = 1}^{k} \mathop \sum \limits_{{x_{j} \in S_{i} }} \left| {x_{j} - \mu_{i} } \right|^{2} $$

We used Python Anaconda (Python version 3.7, https://www.anaconda.com (accessed on 10 August 2021); Anaconda Inc., Austin, TX, USA) and with Scikit-learn 0.24 (sklearn.cluster.KMeans; https://scikit-learn.org/stable/index.html (accessed on 10 August 2021) for clustering. For the number of clusters, the elbow method was used. The elbow method monitors the results while sequentially increasing the number of clusters, and yields a point where diminishing returns are no longer worth the addition cost (Additional File 1)^40^.

### Statistical analysis

Categorical variables are presented as number (percentage), and continuous variables are presented as mean (± SD). Pearson’s chi-square test was used for comparing categorical variables, and Student’s *t*-test was used to compare continuous variables. Kaplan‒Meier analysis was performed to assess the cumulative mortality for 90 days, and Kaplan‒Meier curves were compared using the log-rank test. Univariate and multivariable Cox proportional hazard regression analyses were performed to determine the prognostic factors of 90-day mortality. Significant variables in the univariate analyses (*p* < 0.05) were included in the multivariable analysis. Statistical analyses were performed using the Statistical Package for the Social Sciences (SPSS) version 26.0 (IBM Corporation, Armonk, NY, USA), and P values less than 0.05 were considered statistically significant.

### Ethics approval and consent to participate

This retrospective study protocol was approved by the institutional review board of Chuncheon Sacred Hospital (CHUNCHEON 2021-09-004). The need for obtaining informed consent was waived due to the retrospective nature of the study.

## Supplementary Information

Supplementary Information 1.

Supplementary Information 2.

## Data Availability

The datasets used and analyzed during the current study are available from the corresponding author on reasonable request.

## References

[1] Singer M (2016). The third international consensus definitions for sepsis and septic shock (Sepsis-3). JAMA.

[2] Kaukonen KM, Bailey M, Pilcher D, Cooper DJ, Bellomo R (2015). Systemic inflammatory response syndrome criteria in defining severe sepsis. N. Engl. J. Med..

[3] Clemmer TP (1992). Hypothermia in the sepsis syndrome and clinical outcome. The methylprednisolone severe sepsis study group. Crit. Care Med..

[4] Marik PE, Zaloga GP (2000). Hypothermia and cytokines in septic shock. Norasept II Study Investigators. North American study of the safety and efficacy of murine monoclonal antibody to tumor necrosis factor for the treatment of septic shock. Intensive Care Med..

[5] Park S (2020). Normothermia in patients with sepsis who present to emergency departments is associated with low compliance with sepsis bundles and increased in-hospital mortality rate. Crit. Care Med..

[6] Inghammar M, Sunden-Cullberg J (2020). Prognostic significance of body temperature in the emergency department vs the ICU in patients with severe sepsis or septic shock: A nationwide cohort study. PLoS ONE.

[7] Rumbus Z (2017). Fever is associated with reduced, hypothermia with increased mortality in septic patients: A meta-analysis of clinical trials. PLoS ONE.

[8] Martin-Loeches I (2019). Risk factors for mortality in elderly and very elderly critically ill patients with sepsis: a prospective, observational, multicenter cohort study. Ann. Intensive Care.

[9] Shimazui T (2020). Significance of body temperature in elderly patients with sepsis. Critical care (London, England).

[10] Leligdowicz A, Matthay MA (2019). Heterogeneity in sepsis: New biological evidence with clinical applications. Crit. Care (London, England).

[11] Knox DB, Lanspa MJ, Kuttler KG, Brewer SC, Brown SM (2015). Phenotypic clusters within sepsis-associated multiple organ dysfunction syndrome. Intensive Care Med..

[12] Bhavani SV (2019). Identifying novel sepsis subphenotypes using temperature trajectories. Am. J. Respir. Crit. Care Med..

[13] Zhang Z (2020). Deep learning-based clustering robustly identified two classes of sepsis with both prognostic and predictive values. EBioMedicine.

[14] Leon LR (2004). Hypothermia in systemic inflammation: Role of cytokines. Front. Biosci..

[15] Rumbus Z, Garami A (2017). Fever, hypothermia, and mortality in sepsis: Comment on: Rumbus Z, Matics R, Hegyi P, Zsiboras C, Szabo I, Illes A, Petervari E, Balasko M, Marta K, Miko A, Parniczky A, Tenk J, Rostas I, Solymar M, Garami A. Fever is associated with reduced, hypothermia with increased mortality in septic patients: A meta-analysis of clinical trials. PLoS ONE.

[16] Romanovsky AA, Székely M (1998). Fever and hypothermia: Two adaptive thermoregulatory responses to systemic inflammation. Med. Hypotheses.

[17] Chester JG, Rudolph JL (2011). Vital signs in older patients: Age-related changes. J. Am. Med. Dir. Assoc..

[18] Kenney WL, Munce TA (2003). Invited review: Aging and human temperature regulation (Bethesda, Md.: 1985). J. Appl. Physiol..

[19] Sund-Levander M, Grodzinsky E (2009). Time for a change to assess and evaluate body temperature in clinical practice. Int. J. Nurs. Pract..

[20] Collins KJ (1977). Accidental hypothermia and impaired temperature homoeostasis in the elderly. BMJ.

[21] Daniels F, Baker PT (1961). Relationship between body fat and shivering in air at 15 C. J. Appl. Physiol..

[22] Falk B, Bar-Or O, Smolander J, Frost G (1994). Response to rest and exercise in the cold: Effects of age and aerobic fitness (Bethesda, Md.: 1985). J. Appl. Physiol..

[23] Kwiatkowski D (1989). Febrile temperatures can synchronize the growth of *Plasmodium falciparum* in vitro. J. Exp. Med..

[24] Mackowiak PA, Marling-Cason M, Cohen RL (1982). Effects of temperature on antimicrobial susceptibility of bacteria. J. Infect. Dis..

[25] Boonmee P, Ruangsomboon O, Limsuwat C, Chakorn T (2020). Predictors of mortality in elderly and very elderly emergency patients with sepsis: A retrospective study. West. J. Emerg. Med..

[26] Tiruvoipati R (2010). Hypothermia predicts mortality in critically ill elderly patients with sepsis. BMC Geriatr..

[27] Mackowiak PA, Wasserman SS, Levine MM (1992). A critical appraisal of 98.6 degrees F, the upper limit of the normal body temperature, and other legacies of Carl Reinhold August Wunderlich. JAMA.

[28] O'Grady NP (2008). Guidelines for evaluation of new fever in critically ill adult patients: 2008 update from the American College of Critical Care Medicine and the Infectious Diseases Society of America. Crit. Care Med..

[29] Peres Bota D, Lopes Ferreira F, Mélot C, Vincent JL (2004). Body temperature alterations in the critically ill. Intensive Care Med..

[30] Wiewel MA (2016). Risk factors, host response and outcome of hypothermic sepsis. Crit. Care (London, England).

[31] Keating HJ, Klimek JJ, Levine DS, Kiernan FJ (1984). Effect of aging on the clinical significance of fever in ambulatory adult patients. J. Am. Geriatr. Soc..

[32] Castela Forte J (2021). Identifying and characterizing high-risk clusters in a heterogeneous ICU population with deep embedded clustering. Sci. Rep..

[33] Rodríguez A (2021). Deploying unsupervised clustering analysis to derive clinical phenotypes and risk factors associated with mortality risk in 2022 critically ill patients with COVID-19 in Spain. Crit. Care (London, England).

[34] Rhee C (2016). Objective sepsis surveillance using electronic clinical data. Infect. Control Hosp. Epidemiol..

[35] Oh SY (2019). Incidence and outcomes of sepsis in Korea: A nationwide cohort study from 2007 to 2016. Crit. Care Med..

[36] American College of Chest Physicians/Society of Critical Care Medicine Consensus Conference: Definitions for sepsis and organ failure and guidelines for the use of innovative therapies in sepsis. *Crit. Care Med.* **20**, 864–874 (1992). 1597042

[37] Churpek MM, Zadravecz FJ, Winslow C, Howell MD, Edelson DP (2015). Incidence and prognostic value of the systemic inflammatory response syndrome and organ dysfunctions in ward patients. Am. J. Respir. Crit. Care Med..

[38] Likas A, Vlassis N, Verbeek JJ (2003). The global k-means clustering algorithm. Pattern Recogn..

[39] Han J, Pei J, Kamber M (2011). Data Mining: Concepts and Techniques.

[40] Syakur, M. A., Khotimah, B. K., Rochman, E. M. S. & Satoto, B. D. Integration K-means clustering method and elbow method for identification of the best customer profile cluster. *IOP Conference Series Materials Science and Engineering*, 012017 (2018).

