# Supplementary material for: Cluster analysis integrating age and body temperature for mortality in patients with sepsis: a multicenter retrospective study
Source: Sci Rep. 2022 Jan 20;12:1090. doi: 10.1038/s41598-022-05088-z (PMC8776751; doi:10.1038/s41598-022-05088-z)

**Cluster analysis integrating age and body temperature for mortality in patients with sepsis: A multicenter retrospective study**

Moon Seong Baek, MD^1,†^; Jong Ho Kim, MD^2,3,†^; Young Suk Kwon, MD^2,3,^*

^1^ Department of Internal Medicine, Chung-Ang University Hospital, Chung-Ang University College of Medicine, Seoul, Republic of Korea

^2^ Department of Anesthesiology and Pain Medicine, College of Medicine, Hallym University, Chuncheon Sacred Heart Hospital, Chuncheon, Republic of Korea

^3^ Institute of New Frontier Research Team, Hallym University, Chuncheon, South Korea

Additional File 1. Distortion versus number of clusters for the elbow method. Distortion on the graph became flat at a three clusters; thus, this was set as the number of clusters in the analysis.


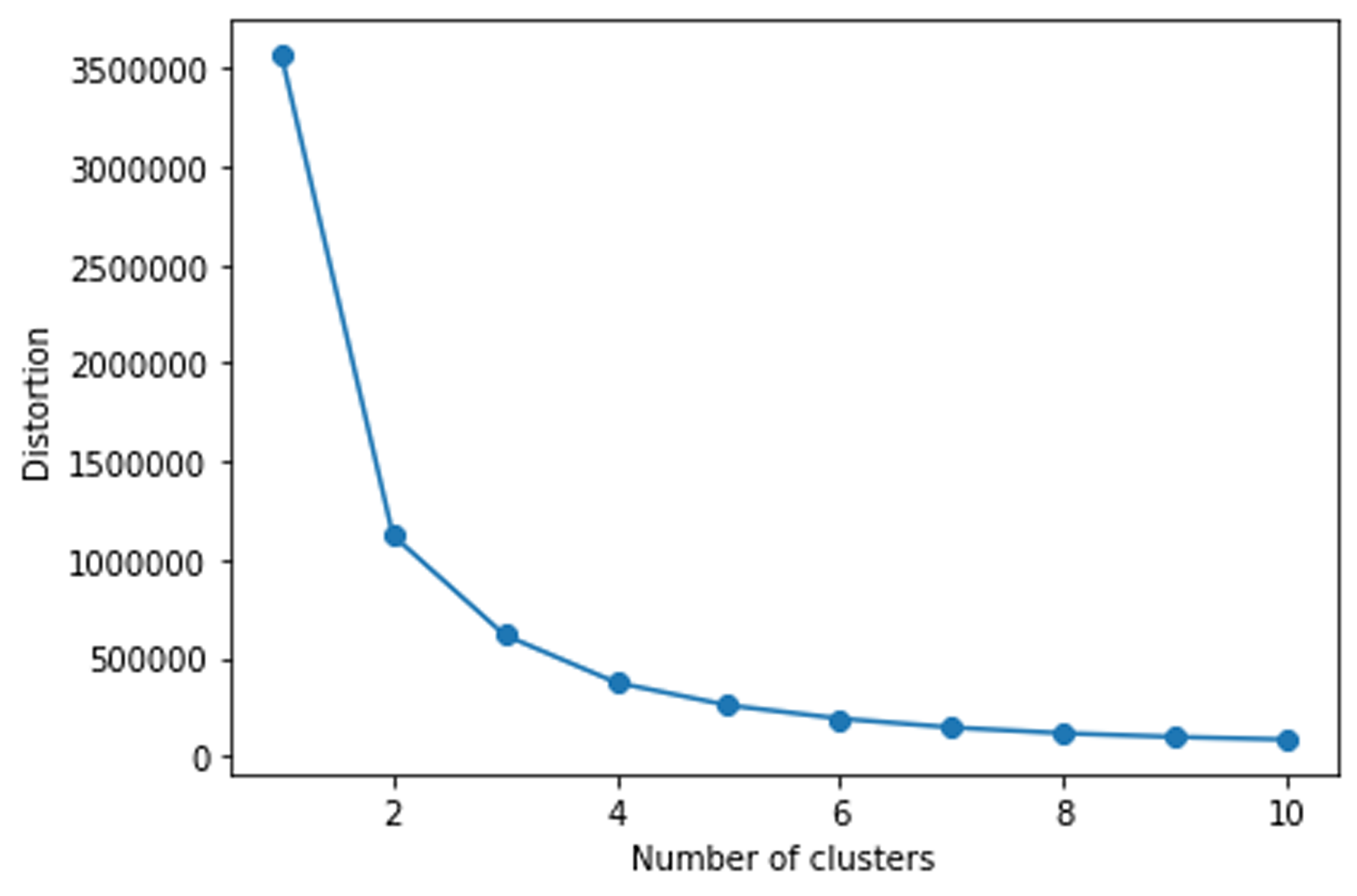

Supplement: Supplementary file 1 — Supplementary Information 1. [file 41598_2022_5088_MOESM1_ESM.docx]
