# Supplementary material for: Cluster analysis integrating age and body temperature for mortality in patients with sepsis: a multicenter retrospective study
Source: Sci Rep. 2022 Jan 20;12:1090. doi: 10.1038/s41598-022-05088-z (PMC8776751; doi:10.1038/s41598-022-05088-z)
Supplement: Supplementary file 2 — Supplementary Information 2. [file 41598_2022_5088_MOESM2_ESM.docx]

**Cluster analysis integrating age and body temperature for mortality in patients with sepsis: A multicenter retrospective study**

Moon Seong Baek, MD^1,†^; Jong Ho Kim, MD^2,3,†^; Young Suk Kwon, MD^2,3,^*

^1^ Department of Internal Medicine, Chung-Ang University Hospital, Chung-Ang University College of Medicine, Seoul, Republic of Korea

^2^ Department of Anesthesiology and Pain Medicine, College of Medicine, Hallym University, Chuncheon Sacred Heart Hospital, Chuncheon, Republic of Korea

^3^ Institute of New Frontier Research Team, Hallym University, Chuncheon, South Korea

Additional File 2. Baseline characteristics of sepsis patients according to three clusters

| Variables | Cluster A  (n=7,520) | Cluster B  (n=4,243) | Cluster C  (n=3,811) | P value |
| --- | --- | --- | --- | --- |
| Age (years) | 79.1 ± 7.3 | 50.9 ± 10.4 | 74.5 ± 9.4 | <0.001 |
| Male sex (%) | 3920 (52.1) | 2979 (70.2) | 2077 (54.5) | <0.001 |
| Body mass index (kg/m^2^) | 21.5 ± 4.0 | 22.6 ± 4.5 | 22.4 ± 4.1 | <0.001 |
| SIRS | 1.7 ± 0.9 | 1.8 ± 1.0 | 2.4 ± 1.0 | <0.001 |
| qSOFA | 1.6 ± 0.8 | 1.4 ± 0.8 | 1.5 ± 0.8 | <0.001 |
| SOFA score | 6.5 ± 3.3 | 6.7 ± 3.5 | 6.3 ± 3.2 | <0.001 |
| APACHE II score | 20.8 ± 7.0 | 18.5 ± 7.4 | 19.0 ± 6.2 | <0.001 |
| Charlson comorbidity index | 6.2 ± 2.3 | 3.2 ± 2.5 | 5.8 ± 2.5 | <0.001 |
| Comorbidities (%) |  |  |  |  |
| Hypertension | 5071 (67.4) | 1533 (36.1) | 2496 (65.5) | <0.001 |
| Diabetes | 3059 (40.7) | 1413 (33.3) | 1651 (43.3) | <0.001 |
| Cardiac disease | 2853 (37.9) | 872 (20.6) | 1117 (29.3) | <0.001 |
| Chronic lung disease | 1693 (22.5) | 484 (11.4) | 885 (23.2) | <0.001 |
| Chronic renal disease | 1330 (17.7) | 549 (12.9) | 573 (15.0) | <0.001 |
| Chronic liver disease | 589 (7.8) | 1148 (27.1) | 369 (9.7) | <0.001 |
| Cerebrovascular disease | 2538 (33.8) | 764 (18.0) | 1362 (35.7) | <0.001 |
| Solid cancer | 1643 (21.8) | 702 (16.5) | 944 (24.8) | <0.001 |
| Hematologic malignancy | 125 (1.7) | 61 (1.4) | 66 (1.7) | 0.530 |
| Body temperature (ºC) | 36.5 ± 0.7 | 36.6 ± 1.0 | 38.2 ± 0.8 | <0.001 |
| < 36 (ºC) | 937 (12.5) | 567 (13.4) | 0 (0.0) | <0.001 |
| 36–38 (ºC) | 6583 (87.5) | 3373 (79.5) | 1499 (39.3) |  |
| > 38 (ºC) | 0 (0.0) | 303 (7.1) | 2312 (60.7) |  |
| Septic shock (%) | 2160 (28.7) | 1281 (30.2) | 1104 (29.0) | <0.001 |
| Mechanical ventilation (%) | 2640 (35.1) | 1709 (40.3) | 886 (23.2) | <0.001 |
| CRRT (%) | 1073 (14.3) | 790 (18.6) | 332 (8.7) | <0.001 |
| Vasopressor use (%) | 2705 (36.0) | 1558 (36.7) | 1439 (37.8) | <0.001 |
| Corticosteroid use (%) | 1263 (16.8) | 644 (15.2) | 607 (15.9) | 0.067 |
| Transfusion (%) | 1400 (18.6) | 1241 (29.2) | 488 (12.8) | <0.001 |
| Combination antibiotic therapy (%) | 4636 (61.6) | 2251 (53.1) | 2734 (71.7) | <0.001 |
| Length of stay (day) | 18.2 ± 20.9 | 18.3 ± 24.7 | 19.1 ± 21.1 | 0.765 |
| ICU stay (day) | 9.4 ± 13.1 | 9.7 ± 16.9 | 8.6 ± 12.5 | 0.387 |

Values are presented as mean ± SD, or n (%).

SIRS, systemic inflammatory response syndrome; SOFA, sequential organ failure assessment; APACHE, acute physiology and chronic health evaluation; CRRT, continuous renal replacement therapy; ICU, and intensive care unit.
